# 113 Predictors of Pain and Open Wounds Following CO2 Laser Management of Burn Scars

**DOI:** 10.1093/jbcr/iraf019.113

**Published:** 2025-04-01

**Authors:** Madysen Johnson, Janet Ashley, Kim Walker, Lauren Benjamin, Jessica Nye, David Wainwright

**Affiliations:** University of Texas Health Science Center at Houston; University of Texas Health Science Center at Houston; John S. Dunn Burn Center; John S. Dunn Burn Center; University of Texas Health Science Center at Houston; John S. Dunn Burn Center

## Abstract

**Introduction:**

The CO2 laser can be beneficial in scar management, but there is a risk of pain and open wounds. This study aims to identify predictors of pain and wound formation based on treatment details and patient reported outcomes.

**Methods:**

Patients undergoing UltraPulse Laser treatment for burn scarring were eligible for inclusion. The primary outcomes were the presence of pain or wound formation. Secondary outcomes were pain severity, wound severity, analgesic use and return to normal scar management. To assess these outcomes, an institutional developed questionnaire was completed after each laser session. Additional data collection included demographics, burn injury details and details of laser treatment. Statistical analyses were performed using R studio (Posit, PBC). Statistical significance was denoted by a p-value < 0.05.

**Results:**

Sixty-nine patients undergoing 337 treatments met inclusion criteria. Completed evaluations from 235 sessions were available for analysis. The average patient age was 46.0 years (range 23-77) with almost almost 2/3 males (45 vs 24). Average burn size was 21.4% (range 1-89%). The average Fitzpatrick skin type was 3.58 (range 2-5). Laser treatment averages were 4.5 sessions, 601.86 cm2 treated per session after 543.8 days after injury. Patients reported pain following 67.6% of treatments, ranging from mild(47.1%) to severe (12.6%). In patients experiencing pain, medication was utilized after 85% of treatments, with 61% requiring narcotics. Wounds were reported after 40% of treatments, 95.8% being superficial. Topical scar management techniques were resumed at a median of 4.4 days (moisturizer) and 5.2 days (garments). On univariate analysis, race, gender, age and treatment location/size did not impact pain experience. When pain level groups were evaluated, higher pain levels were significantly associated with earlier treatment after burn (427 vs 608 days), session number (3.3 vs 4.8), laser density (4.2 vs 3.3) and wound severity (.75 vs.32). Wound formation generated similar values for these variables and both led to delays in resuming standard scar modalities.

**Conclusions:**

Conclusion: After laser treatment, pain and open wounds were generally mild, minor and well tolerated. Timing after burn injury, session number and laser density are potentially predictors of pain and wound formation following laser treatment. Further goals will include assessment of the patient experience with variation in other injury and management factors.

**Applicability of Research to Practice:**

Applicability to Clinical Practice: Identifies treatment factors that can influence patient outcomes following laser scar management.

**Funding for the Study:**

N/A

